# Dietary factors and lung cancer risk in Japanese: with special reference to fish consumption and adenocarcinomas

**DOI:** 10.1054/bjoc.2001.1722

**Published:** 2001-05

**Authors:** T Takezaki, K Hirose, M Inoue, N Hamajima, Y Yatabe, T Mitsudomi, T Sugiura, T Kuroishi, K Tajima

**Affiliations:** Division of Epidemiology and Prevention, Aichi Cancer Center Research Institute, 1-1 Kanokoden, Chikusa-ku, Nagoya 464-8681; Pathology and Clinical Laboratories; Thoracic Surgery; Departments of Internal Medicine, Aichi Cancer Center Hospital, Nagoya, Japan

**Keywords:** fish consumption, lung neoplasms, risk, adenocarcinoma

## Abstract

To investigate risk modification for lung cancer with diet in Japanese, we conducted a hospital-based case–control study and evaluated variation in influence with the histological type. We recruited 367 male and 240 female cases with adenocarcinomas, and 381 male and 57 female cases with squamous cell and small cell carcinomas. Controls comprised 2964 male and 1189 female cancer-free outpatients matched for sex and age with the cases. Odds ratios (ORs) and their 95% confidence intervals (CIs) for lung cancer were calculated with adjustment for potential confounding factors, using an unconditional logistic model. We found decreased ORs for adenocarcinomas in both males (OR = 0.51, 95% CI 0.31–0.84) and females (OR = 0.48, 95% CI 0.24–0.94) who consumed cooked/raw fish, but not dried/salted fish at the highest quartile frequency, compared with the lowest. Soybean curd consumption was associated with a decreased OR for female adenocarcinomas. Decreased ORs for squamous cell and small cell carcinomas were observed in males with frequent consumption of raw and green vegetables, fruit and milk, but consumption of carrot, pumpkin, egg and coffee was associated with increased ORs. This study suggests cooked/raw fish consumption lowers the risk of adenocarcinoma of the lung in Japanese. © 2001 Cancer Research Campaign http://www.bjcancer.com


British Journal of Cancer (2001) 84(9), 1199–1206
© 2001 Cancer Research Campaign
doi: 10.1054/ bjoc.2001.1722, available online at http://www.idealibrary.com on IDEAL®

http://www.bjcancer.com

### Dietary factors and lung cancer risk in Japanese: with special reference to fish consumption and adenocarcinomas

**T Takezaki[1], K Hirose[1], M Inoue[1], N Hamajima[1], Y Yatabe[2], T Mitsudomi[3], T Sugiura[4], T Kuroishi[1] and K Tajima[1]**

[1]Division of Epidemiology and Prevention, Aichi Cancer Center Research Institute, 1-1 Kanokoden, Chikusa-ku, Nagoya 464-8681; [2]Pathology and Clinical Laboratories; [3]Thoracic Surgery; [4]Departments of Internal Medicine, Aichi Cancer Center Hospital, Nagoya, Japan

**Summary** To investigate risk modification for lung cancer with diet in Japanese, we conducted a hospital-based case–control study and evaluated variation in influence with the histological type. We recruited 367 male and 240 female cases with adenocarcinomas, and 381 male and 57 female cases with squamous cell and small cell carcinomas. Controls comprised 2964 male and 1189 female cancer-free outpatients matched for sex and age with the cases. Odds ratios (ORs) and their 95% confidence intervals (CIs) for lung cancer were calculated with adjustment for potential confounding factors, using an unconditional logistic model. We found decreased ORs for adenocarcinomas in both males (OR = 0.51, 95% CI 0.31–0.84) and females (OR = 0.48, 95% CI 0.24–0.94) who consumed cooked/raw fish, but not dried/salted fish at the highest quartile frequency, compared with the lowest. Soybean curd consumption was associated with a decreased OR for female adenocarcinomas. Decreased ORs for squamous cell and small cell carcinomas were observed in males with frequent consumption of raw and green vegetables, fruit and milk, but consumption of carrot, pumpkin, egg and coffee was associated with increased ORs. This study suggests cooked/raw fish consumption lowers the risk of adenocarcinoma of the lung in Japanese. © 2001 Cancer Research Campaign http://www.bjcancer.com

**Keywords**: fish consumption; lung neoplasms; risk; adenocarcinoma

Lung cancer is the leading cause of death among malignancies in Japan (Statistics and Information Department, Ministry of Health and Welfare, 2000). Its age-adjusted mortality and incidence rates, however, are still less than two-thirds of those in the US and the UK (Parkin et al, 1999; Pisani et al, 1999). Wynder and Hoffmann have pointed out the importance of dietary factors as underlying reasons for the lower lung cancer death rates observed in Japan, in addition to age at onset of cigarette smoking, personal smoking behaviour and the make-up of cigarettes (Wynder and Hoffmann, 1994). Japanese specific dietary factors that may protect against cancer development include frequent consumption of soybean products, green tea and fish. We here examine the hypothesis that intake of these foods modifies the risk of lung cancer in Japanese.

A number of studies have revealed negative associations between fish consumption and lung cancer risk (Koo, 1988; Pierce et al, 1989; Veierod et al, 1997). Furthermore, an inverse correlation between fish consumption and male lung cancer mortality was observed in a recent international ecological study (Zhang et al, 2000). However, a protective effect of fish consumption with regard to lung cancer risk is not yet conclusive (World Cancer Research Fund, 1997), and one problem is the variety of forms of lung neoplasia. We are aware of no published reports focused on the risk for lung cancer with fish consumption by histological type. N-3 polyunsaturated fatty acids (PUFA) present in fish oil, eicosapentaenoic acid (EPA) and docosahexaenoic acid (DHA), exert inhibitory effects on development of human lung mucoepidermoid and other carcinomas (Karmali et al, 1984; de Bravo et al, 1991; Ling et al, 1991; Rose and Connoclly, 1991; Singh et al, 1997).

Experimental studies have revealed protective effects of isoflavones from soybeans and epigallocatechin gallate from green tea against lung cancer development (Kinlen et al, 1988; Messina et al, 1994; Fujiki et al, 1998; Lian et al, 1999). Several studies in Japan and China have shown risk reduction of lung cancer with consumption of soybean products (Koo, 1988; Wakai et al, 1999a) and green tea (Kinlen et al, 1988; Ohno et al, 1995), but again data for histological types are limited.

To investigate the association between food consumption and lung cancer risk with reference to histopathological diagnosis, we here conducted a hospital-based case–control study.

*Received 4 September 2000*
*Revised 23 January 2001*
*Accepted 24 January 2001*

*Correspondence to:* T Takezaki

### SUBJECTS AND METHODS

### Study subjects and data collection

Since 1988, a self-administered questionnaire has been completed by first-visit outpatients before undergoing medical examinations at Aichi Cancer Center Hospital in Nagoya, Japan (Hospital-Based Epidemiologic Research Program at Aichi Cancer Center: HERPACC). Details of the questionnaire and data collection procedures have been described elsewhere (Inoue et al, 1995; Tajima et al, 2000). In brief, an expert interviewer checks all written responses at the time of collection within the first-visit day. Included are questions on demographic information, prior medical history, family history, smoking and drinking habits, general health condition, reproduction, and food intake before the onset of the current symptoms. Questions regarding green tea and coffee consumption were added in 1990. The data are annually linked to the hospital cancer-registry system to obtain information on confirmed diagnosis one year after the first visit. Between 1988 and 1997, questionnaires were handed out to 67 854 (91.3%) first-visit outpatients ($n$ = 74 280), and collected with a high response rate (98.6%).

From 1988 to 1997, 1474 cases presented at the Hospital Departments of Internal Medicine or Thoracic Surgery, Aichi Cancer Center Hospital, as first-visit outpatients, and were histologically diagnosed as having primary cancer of the lung (ICD9-162 or ICD10-C34). The histological classification was performed according to the criteria of the World Health Organization (World Health Organization, 1981). Operations were performed in 658 cases, and the diagnosis in the remaining 816 cases was confirmed by biopsy and cytological examination. Of the total, 1212 cases answered our questionnaire. The remaining 263 were hospitalized without taking outpatient service, or were unable to answer the questions because of severe symptoms. The patients with large cell carcinomas of the lung were not included in the cases, because their number (67 males and 11 females) was not sufficient for analyses. We also excluded patients who had missing values for smoking status or fish consumption. Finally, 748 male and 297 female patients aged 40 to 79 years who were histologically diagnosed as having adenocarcinomas, squamous cell carcinomas, or small cell carcinomas of the lung were eligible as study subjects.

The controls were outpatients confirmed to be cancer-free by diagnostic procedures at Aichi Cancer Center Hospital between 1988 and 1997. Also excluded were those with a prior medical history of cancer, with unrecorded smoking status or fish consumption details, or who had been referred from doctors, a group with a high potential for having precancerous diseases. Of the remaining 10 418 male and 23 841 female outpatients, we, then, randomly selected 4 controls per one case after matching sex and age (within 5 years). Finally, 2964 males and 1189 females were eligible for the control group.

### Data analysis

We defined current smokers as persons who had smoked for one year or more, or had quit smoking less than one year before the questionnaire study, ex-smokers as those who had smoked for one year or more and quit smoking one year or more before the study, and never-smokers as those who never smoked or smoked for less than one year. We similarly divided smoking status of husbands into 3 categories as never smokers, former smokers, and current smokers. Females with no husband were included in the category of never smokers for smoking status of husband. We categorized current and prior occupations into 2 groups, blue-collar workers, and white-collar workers, because previous studies reported a positive association between the former and risk for lung cancer (Blot and Fraumeni, 1996). Medical staffs were included as blue-collar workers and housewives as white-collar workers. With regard to personal history, prior lung diseases taken into account included bronchitis, pneumonia, pleurisy, lung abscess formation, pneumothorax, interstitial pneumonia, and chronic obstructive lung diseases.

3 dietary habits, 2 beverages and 24 food items were analysed as factors. Beverages and food items were divided into 4 or 5 groups according to frequency of consumption before the onset of the current symptoms. These groups were further divided into quartile subgroups, each comprising approximately 25% of the controls. Cooked/raw fish was considered separately from dried/salted fish.

We combined cases with squamous cell carcinoma and small cell carcinoma, because the small number of male nonsmoking cases (1 patient with squamous cell carcinoma and 2 patients with small cell carcinoma) led to an unstable estimation of odds ratios (ORs) and their 95% confidence intervals (CIs).

### Statistical analysis

For data analyses, ORs and their 95% CIs of all items for cases and controls were calculated, using the unconditional logistic regression model (Breslow and Day, 1980). $P$ values for trend in the OR were calculated by the chi-square test in the unconditional logistic regression model after making dummy variables, such as 0, 1, 2 and 3. To control for the effects of potential confounding factors, ORs were calculated after adjustment for age (continuous), year and season of the questionnaire study, occupation, smoking status (never, former, current (<20 cigarettes/day) or current (≥20 cigarettes/day)), passive smoking exposure from husbands (never or no husband, former, or current), prior lung diseases, and consumption of green vegetables and meat. The procedure LOGISTIC from the statistical package SAS, version 6.12 (SAS Institute, Cary, NC) was used for the calculations.

### RESULTS

The age distributions of total cases and controls were quite similar, but the average age of adenocarcinoma cases was younger and that of squamous cell and small cell carcinoma cases was older than for the controls (Table 1A, 1B). Current smoking was associated with increased ORs for male and female squamous cell and small cell carcinomas, and male adenocarcinomas, being especially pronounced with squamous cell and small cell carcinomas and heavy smokers (≥20 cigarettes/day). Increased ORs for adenocarcinomas were found for both male and female blue-collar workers. A personal history of prior lung disease was related to increased ORs for adenocarcinomas, as well as squamous cell and small cell carcinomas in both males and females.

In males, we found a decreased OR (0.51; 95% CI = 0.31– 0.84) for adenocarcinomas with the highest quartile consumption of cooked/raw fish, compared with the lowest (Table 2A), the decreasing trend being statistically significant ($P$ = 0.039). The ORs for squamous cell and small cell carcinomas for fish consumption also showed a tendency for decrease but without statistical significance ($P$ = 0.112). Dried/salted fish consumption was not associated with decreased ORs for adenocarcinomas, or squamous cell and small cell carcinomas. Consumption of raw and green vegetables, fruit and milk revealed a significant decreasing trend of ORs for squamous cell and small cell carcinomas, and a decreasing trend of ORs for adenocarcinomas was observed in cases with frequent consumption of green vegetables. In contrast, consumption of carrot, pumpkin, egg and coffee was associated with increased ORs for squamous cell and small cell carcinomas, with statistical significance. Frequent consumption of miso soup increased the ORs for squamous cell and small cell carcinomas, without statistical significance for the trend ($P$ = 0.112). Frequent consumption of pickles of Chinese cabbages also tended to be associated with increased OR for squamous cell and small cell carcinomas.

In females, the highest quartile consumption of cooked/raw fish also decreased the OR (0.48; 95% CI = 0.24 –0.94) for adenocarcinomas, but not for squamous cell and small cell carcinomas

 *© 2001 Cancer Research Campaign*

**Table 1A** Comparison of baseline lung cancer risk factors by histological type in males, Aichi Cancer Center Hospital, 1988–97

| | Referents | Cases | | | | | |
|---|---|---|---|---|---|---|---|
| | | Adenocarcinomas | | | Squamous cell & small cell carcinomas | | |
| | No. | No. | OR[a] | (95% CI) | No. | OR[a] | (95% CI) |
| Age in years | | | | | | | |
| 40–49 | 288 | 54 | | | 18 | | |
| 50–59 | 856 | 121 | | | 93 | | |
| 60–69 | 1099 | 117 | | | 157 | | |
| 70–79 | 721 | 75 | | | 113 | | |
| Total | 2964 | 367 | | | 381 | | |
| Average age in years | 62.1 | 60.2 | | | 64.2 | | |
| Smoking status | | | | | | | |
| Never | 632 | 35 | 1.00 | – | 3 | 1.00 | – |
| Former | 1100 | 86 | 1.52 | (1.00–2.29) | 88 | 15.5 | (4.87–49.3) |
| Current | 1232 | 246 | 3.71 | (2.56–5.37) | 290 | 52.4 | (16.7–164) |
| <20 cigarettes/day | 399 | 48 | 2.25 | (1.42–3.55) | 53 | 25.6 | (7.93–82.6) |
| ≥20 cigarettes/day | 826 | 198 | 4.38 | (3.00–6.40) | 235 | 70.0 | (22.3–220) |
| Occupation | | | | | | | |
| White-collar workers | 2175 | 239 | 1.00 | – | 287 | 1.00 | – |
| Blue-collar workers | 789 | 128 | 1.46 | (1.16–1.85) | 94 | 0.91 | (0.71–1.17) |
| Prior lung diseases | | | | | | | |
| No | 2845 | 342 | 1.00 | – | 342 | 1.00 | – |
| Yes | 119 | 25 | 1.81 | (1.15–2.84) | 39 | 2.65 | (1.81–3.89) |

[a]Adjusted for age (continuous), and year and season of questionnaire study.

**Table 1B** Comparison of baseline lung cancer risk factors by histological type in females, Aichi Cancer Center Hospital, 1988–97

| | Referents | Cases | | | | | |
|---|---|---|---|---|---|---|---|
| | | Adenocarcinomas | | | Squamous cell & small cell carcinomas | | |
| | No. | No. | OR[a] | (95% CI) | No. | OR[a] | (95% CI) |
| Age in years | | | | | | | |
| 40–49 | 220 | 46 | | | 9 | | |
| 50–59 | 352 | 76 | | | 12 | | |
| 60–69 | 416 | 81 | | | 23 | | |
| 70–79 | 201 | 37 | | | 13 | | |
| Total | 1189 | 240 | | | 57 | | |
| Average age in years | 59.5 | 59.0 | | | 61.6 | | |
| Smoking status | | | | | | | |
| Never | 1043 | 209 | 1.00 | – | 20 | 1.00 | – |
| Former | 45 | 8 | 0.89 | (0.41–1.93) | 5 | 6.33 | (2.14–18.7) |
| Current | 101 | 23 | 1.13 | (0.70–1.84) | 32 | 18.4 | (9.94–34.1) |
| <20 cigarettes/day | 69 | 11 | 0.82 | (0.43–1.59) | 18 | 14.6 | (7.26–29.5) |
| ≥20 cigarrettes/day | 32 | 12 | 1.76 | (0.88–3.50) | 14 | 26.4 | (11.7–59.8) |
| Occupation | | | | | | | |
| White-collar workers | 1062 | 198 | 1.00 | – | 48 | 1.00 | – |
| Blue-collar workers | 127 | 42 | 1.85 | (1.26–2.72) | 9 | 1.67 | (0.79–3.52) |
| Prior lung diseases | | | | | | | |
| No | 1154 | 223 | 1.00 | – | 50 | 1.00 | – |
| Yes | 35 | 17 | 2.45 | (1.33–4.49) | 7 | 4.61 | (1.91–11.1) |

[a]Adjusted for age (continuous), and year and season of questionnaire study.

(Table 2B). In contrast, an increased OR (2.83; 95% CI = 1.02–7.82) for squamous cell and small cell carcinoma was found in females who consumed dried/salted fish frequently ($P$ = 0.056 for trend). Frequent consumption of soybean curds lowered the ORs for adenocarcinomas ($P$ = 0.021 for trend), but not for squamous cell and small cell carcinomas ($P$ = 0.519 for trend). Decreased ORs with vegetable and fruit consumption were not apparent for either histological type, except for decrease in ORs for adenocarcinomas with carrot consumption. Frequent consumption of pickled Chinese cabbage revealed an increasing trend of the ORs for squamous cell and small cell carcinomas ($P$ = 0.033).

### DISCUSSION

The present study revealed decreased ORs for adenocarcinoma of the lung with frequent consumption of cooked/raw fish in both male and female Japanese.

© 2001 Cancer Research Campaign 

**Table 2A** Estimated odds ratios (ORs) and 95% confidence intervals (CIs) for patients with lung cancer according to food consumption by hitopathological type in males

| | Adenocarcinomas (*n* = 367) | | | | | Squamous cell and small cell carcinomas (*n* = 381) | | | | |
|---|---|---|---|---|---|---|---|---|---|---|
| | Consumption quartile | | | | | Consumption quartile | | | | |
| | 1 (lowest) | 2 | 3 | 4 (highest) | *P* for trend | 1 (lowest) | 2 | 3 | 4 (highest) | *P* for trend |
| | OR[a] | OR[a] (95% CI) | OR[a] (95% CI) | OR[a] (95% CI) | | OR[a] | OR[a] (95% CI) | OR[a] (95% CI) | OR[a] (95% CI) | |
| Vegetables and fruit | | | | | | | | | | |
| Raw vegetables[b] | 1.00 | 1.13 (0.69–1.85) | 1.13 (0.69–1.86) | 1.01 (0.62–1.65) | 0.662 | 1.00 | 1.31 (0.84–2.03) | 0.70 (0.44–1.12) | 0.80 (0.51–1.25) | 0.004 |
| Fruit[b] | 1.00 | 1.17 (0.75–1.85) | 1.02 (0.63–1.65) | 0.98 (0.61–1.58) | 0.383 | 1.00 | 0.88 (0.58–1.34) | 0.81 (0.52–1.26) | 0.61 (0.40–0.95) | 0.007 |
| Green vegetables[c] | 1.00 | 1.21 (0.88–1.67) | 0.90 (0.63–1.28) | 0.77 (0.51–1.15) | 0.041 | 1.00 | 0.95 (0.69–1.30) | 0.90 (0.64–1.27) | 0.49 (0.32–0.74) | 0.002 |
| Carrot[c] | 1.00 | 1.27 (0.97–1.65) | 1.04 (0.71–1.51) | 1.08 (0.67–1.76) | 0.636 | 1.00 | 1.00(0.76–1.31) | 1.61 (1.12–2.31) | 1.49 (0.94–2.36) | 0.020 |
| Pumpkin[c] | 1.00 | 1.23 (0.96–1.59) | 0.87 (0.49–1.53) | 0.84 (0.32–2.16) | 0.681 | 1.00 | 1.20 (0.93–1.57) | 1.67 (1.06–2.62) | 1.23 (0.55–2.77) | 0.036 |
| Pickles of Chinese cabbages[d] | 1.00 | 0.90 (0.67–1.22) | 0.91 (0.68–1.23) | 0.82 (0.58–1.17) | 0.297 | 1.00 | 1.18 (0.85–1.62) | 1.22 (0.89–1.66) | 1.39 (0.99–1.93) | 0.056 |
| Vegetable pickles lightly preserved[d] | 1.00 | 0.79 (0.55–1.11) | 0.91 (0.66–1.26) | 0.71 (0.51–1.01) | 0.125 | 1.00 | 0.54 (0.37–0.80) | 0.70 (0.50–0.99) | 0.93 (0.67–1.28) | 0.865 |
| Meat, milk and egg | | | | | | | | | | |
| Beef[d] | 1.00 | 1.23 (0.82–1.85) | 1.36 (0.92–2.03) | 1.07 (0.62–1.83) | 0.426 | 1.00 | 1.10 (0.74–1.62) | 1.15 (0.78–1.69) | 1.08 (0.64–1.81) | 0.625 |
| Pork[d] | 1.00 | 1.09 (0.77–1.53) | 1.07 (0.75–1.53) | 0.92 (0.54–1.56) | 0.917 | 1.00 | 1.13 (0.80–1.59) | 1.05 (0.73–1.50) | 1.39 (0.85–2.27) | 0.416 |
| Chicken[d] | 1.00 | 0.77 (0.53–1.13) | 0.95 (0.66–1.36) | 0.64 (0.41–1.02) | 0.320 | 1.00 | 0.75 (0.51–1.10) | 0.86 (0.60–1.24) | 0.84 (0.55–1.28) | 0.769 |
| Milk[e] | 1.00 | 0.96 (0.72–1.28) | 0.92 (0.68–1.24) | 0.82 (0.52–1.30) | 0.390 | 1.00 | 0.87 (0.65–1.16) | 0.76 (0.57–1.03) | 0.66 (0.41–1.05) | 0.030 |
| Egg[c] | 1.00 | 1.24 (0.83–1.86) | 1.08 (0.71–1.64) | 1.10 (0.73–1.64) | 0.826 | 1.00 | 1.38 (0.87–2.18) | 1.88 (1.19–2.95) | 1.62 (1.04–2.54) | 0.030 |
| Fish | | | | | | | | | | |
| Cocked/raw fish[c] | 1.00 | 0.83 (0.61–1.13) | 0.89 (0.63–1.25) | 0.51 (0.31–0.84) | 0.039 | 1.00 | 0.94 (0.68–1.29) | 0.95 (0.66–1.36) | 0.63 (0.40–1.01) | 0.112 |
| Dried/salted fish[d] | 1.00 | 0.86 (0.60–1.22) | 1.11 (0.79–1.57) | 0.66 (0.41–1.07) | 0.743 | 1.00 | 1.17 (0.81–1.71) | 1.21 (0.84–1.76) | 1.37 (0.89–2.12) | 0.172 |
| Soybean products | | | | | | | | | | |
| Soybean curds (tofu)[c] | 1.00 | 1.33 (0.95–1.87) | 1.27 (0.88–1.84) | 1.24 (0.83–1.85) | 0.478 | 1.00 | 1.10 (0.78–1.55) | 1.09 (0.76–1.57) | 1.23 (0.84–1.81) | 0.341 |
| Miso soup[e] | 1.00 | 1.93 (0.90–4.14) | 1.65 (0.78–3.51) | 1.40 (0.63–3.11) | 0.346 | 1.00 | 2.24 (0.98–5.11) | 2.40 (1.07–5.38) | 2.50 (1.08–5.79) | 0.112 |
| Beverages | | | | | | | | | | |
| Green tea[f] | 1.00 | 1.06 (0.72–1.57) | 1.11 (0.74–1.66) | 1.33 (0.83–2.15) | 0.250 | 1.00 | 0.99 (0.67–1.47) | 1.17 (0.78–1.73) | 1.08 (0.66–1.75) | 0.443 |
| Coffee[g] | 1.00 | 0.85 (0.61–1.19) | 0.87 (0.60–1.25) | 1.18 (0.80–1.74) | 0.654 | 1.00 | 0.98 (0.70–1.37) | 1.15 (0.80–1.64) | 1.61 (1.09–2.39) | 0.027 |

[a]Adjusted for age (continuous), season and year of visit, occupation, prior lung diseases, smoking (never, former, current [<20 cigarettes/day] and current [≥20 cigarettes/day]), and consumption of green vegetables and meat. Quartiles are [b]almost never; occasionally, 3–4 times/week, and everyday; [c]<1 time/week, 1–2 time/week, 3–4 times/week, and ≥5 times/week; [d]almost never, 1–3 time/month, 1–2 times/week, and ≥3 times/week; [e]almost never, occasionally, 1 time/day, and ≥2 times/day; [f]<1 cup/day, 1–3 cups/day, 4–6 cups/day, and ≥7 cups/day; [g]<1 cup/day, 1 cup/day, 2 cups/day, and ≥3 cups/day.

*© 2001 Cancer Research Campaign*

**Table 2B** Estimated odds ratios (ORs) and 95% confidence intervals (CIs) for patients with lung cancer according to food consumption by hitopathological type in females

| | Adenocarcinomas ($n$ = 367) | | | | | Squamous cell and small cell carcinomas ($n$ = 381) | | | | |
|---|---|---|---|---|---|---|---|---|---|---|
| | Consumption quartile | | | | | Consumption quartile | | | | |
| | 1 (lowest) | 2 | 3 | 4 (highest) | *P* for trend | 1 (lowest) | 2 | 3 | 4 (highest) | *P* for trend |
| | OR[a] | OR[a] (95% CI) | OR[a] (95% CI) | OR[a] (95% CI) | | OR[a] | OR[a] (95% CI) | OR[a] (95% CI) | OR[a] (95% CI) | |
| Vegetables and fruit | | | | | | | | | | |
| Raw vegetables[b] | 1.00 | 0.74 (0.39–1.41) | 0.85 (0.45–1.60) | 0.84 (0.45–1.55) | 0.904 | 1.00 | 0.97 (0.26–3.55) | 2.11 (0.61–7.34) | 1.01 (0.28–3.58) | 0.896 |
| Fruit[b] | 1.00 | 0.71 (0.28–1.82) | 0.78 (0.31–1.97) | 0.68 (0.27–1.70) | 0.536 | 1.00 | 0.50 (0.11–2.31) | 0.34 (0.07–1.60) | 0.49 (0.11–2.13) | 0.668 |
| Green vegetables[c] | 1.00 | 0.83 (0.47–1.45) | 1.09 (0.63–1.88) | 0.64 (0.36–1.15) | 0.230 | 1.00 | 0.83 (0.28–2.42) | 1.00 (0.34–2.89) | 1.37 (0.46–4.09) | 0.310 |
| Carrot[c] | 1.00 | 0.76 (0.49–1.19) | 0.70 (0.43–1.12) | 0.50 (0.29–0.86) | 0.014 | 1.00 | 1.05 (0.46–2.41) | 0.51 (0.19–1.40) | 0.47 (0.16–1.43) | 0.080 |
| Pumpkin[c] | 1.00 | 0.93 (0.67–1.28) | 1.02 (0.66–1.58) | 0.64 (0.28–1.48) | 0.555 | 1.00 | 0.81 (0.40–1.63) | 0.78 (0.30–2.01) | 1.18 (0.32–4.30) | 0.816 |
| Pickles of Chinese cabbages[d] | 1.00 | 0.73 (0.50–1.08) | 0.87 (0.59–1.30) | 0.81 (0.54–1.23) | 0.308 | 1.00 | 1.12 (0.48–2.62) | 1.79 (0.79–4.08) | 2.18 (0.99–4.82) | 0.033 |
| Vegetables pickles lightly preserved[d] | 1.00 | 1.20 (0.77–1.88) | 1.16 (0.75–1.80) | 1.04 (0.68–1.58) | 0.979 | 1.00 | 0.63 (0.20–2.01) | 2.01 (0.80–5.02) | 1.27 (0.50–3.20) | 0.264 |
| Meat, milk and egg | | | | | | | | | | |
| Beef[d] | 1.00 | 1.11 (0.71–1.73) | 1.20 (0.78–1.85) | 1.07 (0.59–1.94) | 0.585 | 1.00 | 0.87 (0.38–1.99) | 0.71 (0.30–1.64) | 1.06 (0.35–3.22) | 0.709 |
| Pork[d] | 1.00 | 1.07 (0.68–1.67) | 1.13 (0.73–1.75) | 0.80 (0.45–1.43) | 0.710 | 1.00 | 0.78 (0.34–1.79) | 0.85 (0.37–1.97) | 0.48 (0.14–1.69) | 0.370 |
| Chicken[d] | 1.00 | 1.18 (0.66–2.11) | 1.06 (0.62–1.83) | 0.94 (0.52–1.70) | 0.536 | 1.00 | 0.66 (0.23–1.87) | 0.99 (0.40–2.44) | 0.75 (0.26–2.16) | 0.900 |
| Milk[e] | 1.00 | 0.81 (0.53–1.25) | 0.96 (0.64–1.43) | 0.67 (0.40–1.11) | 0.314 | 1.00 | 1.34 (0.59–3.05) | 1.12 (0.49–2.60) | 0.87 (0.31–2.46) | 0.789 |
| Egg[c] | 1.00 | 1.40 (0.83–2.37) | 1.15 (0.68–1.95) | 1.05 (0.62–1.78) | 0.463 | 1.00 | 1.21 (0.46–3.19) | 1.16 (0.43–3.12) | 1.06 (0.40–2.76) | 0.965 |
| Fish | | | | | | | | | | |
| Cooked/raw fish[c] | 1.00 | 0.97 (0.66–1.43) | 0.80 (0.51–1.24) | 0.48 (0.24–0.94) | 0.028 | 1.00 | 0.93 (0.44–1.99) | 0.56 (0.22–1.42) | 0.97 (0.28–3.30) | 0.404 |
| Dried/salted fish[d] | 1.00 | 0.67 (0.44–1.01) | 0.66 (0.44–0.99) | 0.79 (0.47–1.34) | 0.274 | 1.00 | 0.99 (0.38–2.55) | 1.15 (0.47–2.84) | 2.83 (1.02–7.82) | 0.056 |
| Soybean products | | | | | | | | | | |
| Soybean curds (tofu)[c] | 1.00 | 0.89 (0.55–1.43) | 0.93 (0.56–1.52) | 0.52 (0.30–0.91) | 0.021 | 1.00 | 3.68 (0.99–13.6) | 2.86 (0.73–11.2) | 3.00 (0.72–12.6) | 0.519 |
| Miso soup[e] | 1.00 | 0.79 (0.36–1.72) | 0.90 (0.42–1.93) | 0.69 (0.30–1.59) | 0.690 | 1.00 | 0.51 (0.12–2.07) | 0.45 (0.11–1.77) | 0.51 (0.11–2.42) | 0.556 |
| Beverages | | | | | | | | | | |
| Green tea[f] | 1.00 | 0.98 (0.58–1.66) | 1.14 (0.68–1.93) | 1.14 (0.61–2.12) | 0.464 | 1.00 | 0.36 (0.14–0.93) | 0.41 (0.16–1.04) | 0.49 (0.17–1.46) | 0.282 |
| Coffee[g] | 1.00 | 0.76 (0.51–1.13) | 0.82 (0.49–1.35) | 1.28 (0.65–2.54) | 0.823 | 1.00 | 0.96 (0.43–2.18) | 0.61 (0.21–1.78) | 0.28 (0.05–1.58) | 0.142 |

[a]Adjusted for age (continuous), season and year of visit, occupation, prior lung diseases, smoking (never, former, current [<20 cigarettes/day] and current [≥20 cigarettes/day]), passive smoking from husband (never, former and current), and consumption of green vegetables and meat. Quartiles are [b]almost never; occasionally, 3–4 times/week, and everyday; [c]<1 time/week, 1–2 time/week, 3–4 times/week, and ≥5 times/week; [d]almost never, 1–3 time/month, 1–2 times/week, and ≥3 times/week; [e]almost never, occasionally, 1 time/day, and ≥2 times/day; [f]<1 cup/day, 1–3 cups/day, 4–6 cups/day, and ≥7 cups/day; [g]<1 cup/day, 1 cup/day, 2 cups/day, and ≥3 cups/day.

© 2001 Cancer Research Campaign

Previous epidemiological studies showed inconsistent results for the association between fish consumption and risk of lung cancer. A study in Norway found a negative association between cod liver oil supplement and lung cancer risk, due to high intake of vitamin A and n-3 PUFA (Veierod et al, 1997). A decreased OR was observed in female nonsmokers of Hong Kong (Koo, 1988) and in males of Australia (Pierce et al, 1989), although the numbers of cases were small. On the other hand, fish consumption was associated with increased OR for lung cancer in males of Yunnan, China, where average consumption of fish in controls was very low (Swanson et al, 1992). Moreover, 2 cohort and 2 case-control studies found no association (Kvale et al, 1983; Knekt et al, 1991; Goodman et al, 1992; Sankaranarayanan et al, 1994). Fish consumption was, however, inversely associated with male lung cancer mortality in countries with high levels of cigarette smoking or animal fat consumption in a recent ecological study (Zhang et al, 2000). Of 36 countries, Japan with lower mortality rate of lung cancer showed higher consumption of cigarette smoking and fish, but lower animal fat intake.

Cyclo oxygenase-2 (COX-2) is a key inducible enzyme in arachidonic acid metabolism, and experimental and pathological studies have provided evidence of an involvement of COX-2 in carcinogenesis, in addition to inflammatory reactions (Vane, 1994; Oshima et al, 1996). N-3 PUFA inhibits arachidonic acid metabolism through a mechanism different from that with COX-2 inhibitors, but also modulates tmorigenesis (Karmali, 1987; Vane, 1994). Recent studies found an increase in COX-2 expression in human lung cancer, specifically in adenocarcinomas (Hida et al, 1998; Wolff et al, 1998). The results of our study and previous investigators suggest that effects of fish consumption and COX-2 inhibitors are more pronounced for adenocarcinomas than other histological types.

The fact that dried/salted fish consumption was not associated with any decrease in the OR for lung cancer requires explanation. Fish oil demonstrates rapid degradation due to oxidation and other chemical changes on exposure to air, light and heat (Frankel, 1980; Porter et al, 1995). Dried/salted fish is a preserved food, which is consumed several days at least after processing of raw fish. Decreased concentrations of n-3 PUFAs might therefore be expected. Furthermore, a positive association between preserved food consumption and lung cancer risk has been reported (Koo, 1988; Sankaranarayanan et al, 1994; Wakai et al, 1999a). Oxidized n3-PUFA and various contaminants in preserved dried/salted fish might have been linked to the increased OR observed for female cases with squamous cell and small cell carcinomas. An increased OR was also observed for those who consumed another preserved food, pickled Chinese cabbage. Smoking was not associated, because smoking habit was not related to food consumption in female controls and similar results were obtained with and without adjustment for smoking (data not shown).

Previous studies have demonstrated an inverse association between soybean consumption and lung cancer risk in non-smoking female cases with adenocarcinomas or large cell carcinomas in Hong Kong (Koo, 1988), in male cases of Yunnan (Swanson et al, 1992), in male and female cases of Shanghai (Ershow et al, 1990), and in male and female cases with squamous cell carcinoma in Japan (Wakai et al, 1999a). The present results were partially concordant with the first study. Isoflavones of soybean products have not only anti-oestrogenic and oestrogenic effects, but also non-oestrogenic inhibitory influence on tumour development (messina et al, 1994). Risk reduction for female adenocarcinomas seems to be related to hormone effects. However, the disconcordant results by gender and histological type between studies means that this issue is still controversial.

Miso soup consumption tended in the present study to be associated with an increased risk of squamous cell and small cell carcinomas in males. Miso is not only another source of isoflavones, but also a preserved food that contains nitrosamine precursors (Wakai et al, 1999b). The present result was partially in line with a previous study, showing risk elevation in males and females (Wakai et al, 1999a).

Protective effects of vegetables and fruit consumption have been addressed in numerous studies (World Cancer Research Fund, 1997). Some authors have found obvious risk reduction in smokers (Kvale et al, 1983; Voorrips et al, 2000), concordant with our results for male cases with squamous cell and small cell carcinomas. On the other hand, increased ORs for squamous cell and small cell carcinomas were observed in males who frequently consumed carrot and pumpkin rich in beta-carotene. Recent clinical trials of supplemental beta-carotene did not result risk reduction of lung cancer, but rather an elevation of risk (The Alpha-Tocophenol, Beta Carotene Cancer Prevention Study Group, 1994; Hennekens et al, 1996; Omenn et al, 1996). Intake doses of beta-carotene in these studies were much higher than usual consumption from foods. It is possible that our results still included residual confounding by smoking after the adjustment, because consumption of carrot ($R^2$ = 0.20) and pumpkin ($R^2$ = 0.20) was more strongly correlated to the smoking habit than other vegetables and fruit in our controls. Such a correlation was also observed in coffee drinkers ($R^2$ = 0.25). Some studies have shown a positive association between coffee consumption and lung cancer risk, although the possibility of the residual confounding by smoking remains (World Cancer Research Fund, 1997).

A positive association between dietary fat and lung cancer risk has been reported for Western countries (World Cancer Research Fund, 1997), the association being more consistent for consumption of saturated/animal fat and cholesterol. The present study did not reveal a positive association between meat consumption and lung cancer risk, except for an increased OR for male squamous cell and small cell carcinomas with frequent consumption of eggs rich in cholesterol. To clarify this situation, further investigations of dietary fat by nutrient are required, with adjustment for energy intake.

The decreased OR for squamous cell carcinoma for milk consumption was consistent with previous findings (Kvale et al, 1983). A recent cohort study has also revealed risk reduction for lung cancer mortality with frequent dairy product consumption (Breslow et al, 2000), although other investigators have shown no association or increased risk (Koo, 1988; Goodman et al, 1992; Sankaranarayanan et al, 1994). Dairy products contain linoleic acid, sphingomyelins, butyric acid, retinal, calcium, and vitamin D, that have potential protective effects against tumorigenesis (Breslow et al, 2000).

A limitation of our present study was the lack of information on nutrient intake, especially for n-3 PUFA and n-6 PUFA. Whereas n-3 PUFA inhibits growth and promotion of tumours, n-6 PUFA could stimulate them (Karmali, 1987; DeVries and van Noorden, 1992; Vane, 1994). The n-6 PUFA/n-3 PUFA ratio is, therefore, an important additional factor for tumorigenesis (De Vries and van Noorden, 1992). However, an international ecological study revealed that Japanese consume much more n3-PUFA with a much lower ratio of n6 PUFA/n3 PUFA, compared with Western

© 2001 Cancer Research Campaign countries (Hursting et al, 1990). Major contributors of n-3 PUFA intake are mixed salad oil, vegetable oil, mayonnaise, tofu (soybean curds), chicken eggs and fish among Japanese in the Tokai area (Tokudome et al, 1999), where we recruited our study subjects. These vegetable components and chicken eggs also contribute to n-6 PUFA intake, but this is less so for fish. Therefore, frequent consumption of fish can in this context be positively related not only to n-3 PUFA intake, but also a low ratio of n-6PUFA/n-3 PUFA.

Regarding methodological issues, selection bias needs consideration, since controls were recruited from non-cancer hospital outpatients. However, the distribution of diseases among randomly selected non-cancer controls of Aichi Cancer Center Hospital did not demonstrate any obvious feature in common, with reference to precancerous lesions or tobacco-related conditions (Inoue et al, 1995). Furthermore, to access the quality and validity of our questionnaire, we compared non-cancer ACCH outpatients with subjects randomly selected from the electoral roll (Inoue et al, 1997). We found that, with due consideration of age, sex, and season in the analysis, it is feasible to use non-cancer outpatients as controls.

In conclusion, our findings suggest the consumption of fish rich in n-3 PUFA modifies the risk of development of adenocarcinoma of the lung. This provides support for earlier epidemiological pointers to an involvement of n-3 PUFA in carcinogenesis. The relatively lower mortality rate of lung cancer in Japan might thus be at least partly attributable to higher consumption of fish.

### ACKNOWLEDGEMENTS

This study was supported in part by a Grant-in-Aid for Cancer Research and the Comprehensive 10-year Strategy for Cancer Control from the Ministry of Health and Welfare of Japan. We are grateful to Mss Hiroko Fujikura, Yukiko Yamauchi, Etsuko Kimura and Michiko Takasaki for their data collection and preparation; and Dr Suketami Tominaga, Aichi Cancer Center Research Insitute, for critical suggestions regarding the study design.

### REFERENCES

Blot WJ and Fraumeni JF Jr (1996) Cancers of the lung and pleura. In: Schottenfeld D and Fraumeni JF Jr. (eds) *Cancer Epidemiology and Prevention*, 2nd edn, pp 637–665, Oxford University Press: New York

Breslow NE and Day NE (1980) *Statistical Methods in Cancer Research*. Vol. 1. IARC Scientific Publications: Lyon

Breslow RA, Graubard BI, Sinha R and Subar AF (2000) Diet and lung cancer mortality: a 1987 National Health Interview Survey cohort study. *Cancer Causes Control* **11**: 419–431

de Bravo MG, de Antueno RJ, Toledo J, De Tomas ME, Mercuri OF and Quintans C (1991) Effects of an eicosapentaenoic and docosahexaenoic acid concentrate on a human lung carcinoma grown in nude mice. *Lipids* **26**: 866–870

De Vries CE and van Noorden CJ (1992) Effects of dietary fatty acid composition on tumor growth and metastasis. *Anticancer Res* **12**: 1513–1522

Ershow AG, gao YT, Zheng W and Blot WJ (1990) Diet effects on lung cancer risk of Chinese men and women. *FASEB J* **4**: 1041

Frankel EN (1980) Lipid oxidation. *Prog Lipid Res* **19**: 1–22

Fujiki H, Suganuma M, Okabe S, Sueoka N, Komori A, Sueoka E, Kozu T, Tada Y, Suga K, Imai K and Nakachi K (1998) Cancer inhibition by green tea. *Mutat Res* **402**: 307–310

Goodman MT, Hankin JH, Wilkens LR and Kolonel LN (1992) High-fat foods and the risk of lung cancer. *Epidemiology* **3**: 288–299

Hennekens CH, Buring JE, Manson JE, Stampfer M, Rosner B, Cook NR, Belanger C, LaMotte F, Gaziano JM, Ridker PM, Willett W and Peto R (1996) Lack of effect of long-term supplementation with beta carotene on the incidence of malignant neoplasms and cardiovascular disease. *N Engl J Med* **334**: 1145–1149

Hida T, Yatabe Y, Achiwa H, Muramatsu H, Kozaki K, Nakamura S, Ogawa M, Mitsudomi T, Sugiura T and Takahashi T (1998) Increased expression of cyclooxygenase 2 occurs frequently in human lung cancers, specifically in adenocarcinomas. *Cancer Res* **58**: 3761–3764

Hursting SD, Thornquist M and Henderson MM (1990) Types of dietary fat and the incidence of cancer at five sites. *Prev Med* **19**: 242–253

Inoue M, Tajima K, Hirose K, Hamajima N, Takezaki T, Hirai T, Kato T and Ohno Y (1995) Subsite-specific risk factors for colorectal cancer: a hospital-based case-control study in Japan. *Cancer Causes Control* **6**: 14–22

Inoue M, Tajima K, Hirose K, Hamajima N, Takezaki T, Kuroishi T and Tominaga S (1997) Epidemiological features of first-visit outpatients in Japan: comparison with general population and variation by sex, age, and season. *J Clin Epidemiol* **50**: 69–77

Karmali RA (1987) Eicosanoids in neoplasia. *Prev Med* **16**: 493–502

Karmali RA, Marsh J and Fuchs C (1984) Effect of omega-3 fatty acids on growth of a rat mammary tumor. *J Natl Cancer Inst* **73**: 457–461

Kinlen LJ, Willows AN, Goldblatt P and Yudkin J (1988) Tea consumption and cancer. *Br J Cancer* **58**: 397–401

Knekt P, Seppanen R, Jarvinen R, Virtamo J, Hyvonen L, Pukkala E and Teppo L (1991) Dietary cholesterol, fatty acids, and the risk of lung cancer among men. *Nutr Cancer* **16**: 267–275

Koo LC (1988) Dietary habits and lung cancer risk among Chinese females in Hong Kong who never smoked. *Nutr Cancer* **11**: 155–172

Kvale G, Bjelke E and Gart JJ (1983) Dietary habits and lung cancer risk. *Int J Cancer* **31**: 397–405

Lian F, Li Y, Bhuiyan M and Sarkar FH (1999) p53-independent apoptosis induced by genistein in lung cancer cells. *Nutr Cancer* **33**: 125–131

Ling PR, Istfan NW, Lopes SM, Babayan VK, Blackburn GL and Bistrian BR (1991) Structured lipid made from fish oil and medium-chain triglycerides alters tumor and host metabolism in Yoshida-sarcoma-bearing rats. *Am J Clin Nutr* **53**: 1177–1184

Messina MJ, Persky V, Setchell KD and Barnes S (1994) Soy intake and cancer risk: a review of the in vitro and in vivo data. *Nutr Cancer* **21**: 113–131

Ohno Y, Wakai K, Genka K, Ohmine K, Kawamura T, Tamakoshi A, Aoki R, Senda M, Hayashi Y, Nagao K, Fukuma S and Aoki K (1995) Tea consumption and lung cancer risk: a case-control study in Okinawa, Japan. *Jpn J Cancer Res* **86**: 1027–1034

Omenn GS, Goodman GE, Thornquist MD, Balmes J, Cullen MR, Glass A, Keogh JP, Meyskens FL, Valanis B, Williams JH, Barnhart S and Hammar S (1996) Effects of a combination of beta carotene and vitamin A on lung cancer and cardiovascular disease. *N Engl J Med* **334**: 1150–1152

Oshima M, Dinchuk JE, Kargman SL, Oshima H, Hancock B, Kwong E, Trzaskos JM, Evans JF and Taketo MM (1996) Suppression of intestinal polyposis in Apc delta 716 knockout mice by inhibition of cyclooxygenase 2 (COX-2). *Cell* **87**: 803–809

Parkin DM, Pisani P and Ferlay J (1999) Estimates of the worldwide incidence of 25 major cancers in 1990. *Int J Cancer* **80**: 827–841

Pierce RJ, Kune GA, Kune S, Watson LF, Field B, Merenstein D, Hayes A and Irving LB (1989) Dietary and alcohol intake, smoking pattern, occupational risk, and family history in lung cancer patients: results of a case-control study in males. *Nutr Cancer* **12**: 237–248

Pisani P, Parkin DM, Bray F and Ferlay J (1999) Estimates of the worldwide mortality from 25 cancers in 1990. *Int J Cancer* **83**: 18–29

Porter NA, Caldwell SE and Mills KA (1995) Mechanisms of free radical oxidation of unsaturated lipids. *Lipids* **30**: 277–290

Rose DP and Connolly JM (1991) Effects of fatty acids and eicosanoid synthesis inhibitors on the growth of two human prostate cancer cell lines. *Prostate* **18**: 243–254

Sankaranarayanan R, Varghese C, Duffy SW, Padmakumary G, Day NE and Nair MK (1994) A case-control study of diet and lung cancer in Kerala, south India. *Int J Cancer* **58**: 644–649

Singh J, Hamid R and Reddy BS (1997) Dietary fat and colon cancer: modulation of cyclooxygenase-2 by types and amount of dietary fat during the postinitiation stage of colon carcinogenesis. *Cancer Res* **57**: 3465–3470

Statistics and Information Department, Minister's Secretariat, Ministry of Health and Welfare (2000) *Vital Statistics of Japan 1998*, Vol 3. Health and Welfare Statistics Association: Tokyo (in Japanese)

Swanson CA, Mao BL, Li JY, Lubin JH, Yao SX, Wang JZ, Cai SK, Hou Y, Luo QS and Blot WJ (1992) Dietary determinants of lung-cancer risk: results from a case-control study in Yunnan Province, China. *Int J Cancer* **50**: 876–880

Tajima K, Hirose K, Inoue M, Takezaki T, Hamajima N and Kuroishi T (2000) A model of practical prevention for out-patients visiting hospital: the

© 2001 Cancer Research Campaign 

hospital-based epidemiologic research program at Aichi Cancer Center (HERPACC). *Asian Pacific J Cancer Prev* **1**: 35–47
The Alpha-Tocophenol, Beta Carotene Cancer Prevention Study Group (1994) The effect of vitamin E and beta carotene on the incidence of lung cancer and other cancers in male smokers. *N Engl J Med* **330**: 1029–1035
Tokudome Y, Imaeda N, Ikeda M, Kitagawa I, Fujiwara N and Tokudome S (1999) Foods contributing to absolute intake and variance in intake of fat, fatty acids and cholesterol in middle-aged Japanese. *J Epidemiol* **9**: 78–90
Vane J (1994) Towards a better aspirin. *Nature* **367**: 215–216
Veierod MB, Laake P and Thelle DS (1997) Dietary fat intake and risk of lung cancer: a prospective study of 51, 452 Norwegian men and women. *Eur J Cancer Prev* **6**: 540–549
Voorrips LE, Goldbohm RA, Verhoeven DT, van Poppel GA, Sturmans F, Hermus RJ and van den Brandt PA (2000) Vegetable and fruit consumption and lung cancer risk in the Netherlands Cohort Study on diet and cancer. *Cancer Causes Control* **11**: 101–115
Wakai K, Ohno Y, Genka K, Ohmine K, Kawamura T, Tamakoshi A, Lin Y, Nakayama T, Aoki K and Fukuma S (1999a) Risk modification in lung cancer by a dietary intake of preserved foods and soyfoods: findings from a case-control study in Okinawa, Japan. *Lung Cancer* **25**: 147–159
Wakai K, Egami I, Kato K, Kawamura T, Tamakoshi A, Lin Y, Nakayama T, Wada M and Ohno Y (1999b) Dietary intake and sources of isoflavones among Japanese. *Nutr Cancer* **33**: 139–145
Wolff H, Saukkonen K, Anttila S, Karjalainen A, Vainio H and Ristimaki A (1998) Expression of cyclooxygenase-2 in human lung carcinoma. *Cancer Res* **58**: 4997–5001
World Cancer Research Fund in Association with American Institute for Cancer Research (1997) *Lung*. In: *Food, Nutrition and the Prevention of Cancer: a Global Perspective*, World Cancer Research Fund in Association with American Institute for Cancer Research. pp 130–147. American Institute for Cancer Research: Washington DC
World Health Organization (1981) *World Health Organization, Histological Typing of Lung Tumours*, Vol. 1. 2nd ed. International Histological Classification of Tumours No. 1. World Health Organization: Geneva
Wynder EL and Hoffmann D (1994) Smoking and lung cancer: scientific challenges and opportunities. *Cancer Res* **54**: 5284–5295
Zhang J, Temme EH and Kesteloot H (2000) Fish consumption is inversely associated with male lung cancer mortality in countries with high levels of cigarette smoking or animal fat consumption. *Int J Epidemiol* **29**: 615–621

 *© 2001 Cancer Research Campaign*
